# Diet and mitonuclear haplotype interactions affect growth rate in a slime mould

**DOI:** 10.1002/ece3.10508

**Published:** 2023-09-04

**Authors:** Venkatesh Nagarajan‐Radha, Natalie Cordina, Madeleine Beekman

**Affiliations:** ^1^ Behaviour, Ecology and Evolution Lab, School of Life and Environmental Sciences The University of Sydney Camperdown New South Wales Australia

**Keywords:** decision‐making, diet choice, dietary macronutrient ratio, intergenomic interactions, mitonuclear ecology, *Physarum polycephalum*, unicellular eukaryotes

## Abstract

Trait expression in metazoans is strongly influenced by the balance of macronutrients (i.e. protein, carbohydrate and fat) in the diet. At the same time, an individual's genetic background seems to regulate the magnitude of phenotypic response to a particular diet. It needs to be better understood whether interactions between diet, genetic background and trait expression are found in unicellular eukaryotes. A protist—the slime mould, *Physarum polycephalum* can choose diets based on protein‐to‐carbohydrate (P:C) content to support optimal growth rate. Yet, the role of genetic background (variation in the mitochondrial and nuclear DNAs) in mediating growth rate response to dietary P:C ratios in the slime mould is unknown. Here, we studied the effects of interactions between mitochondrial and nuclear DNA haplotypes and diet (i.e. G × G × E interactions) on the growth rate of *P. polycephalum*. A genetic panel of six distinct strains of *P. polycephalum* that differ in their mitochondrial and nuclear DNA haplotypes was used to measure growth rate across five diets that varied in their P:C ratio and total calories. We first determined the strains' growth rate (total biomass and surface area) when grown on a *set* menu with access to a particular diet. We then assessed whether the growth rate of strains increased on a *buffet* menu with access to all diets. Our findings show that the growth rate of *P. polycephalum* is generally higher on diets containing more carbohydrates than protein and that total calories negatively affect the growth rate. Three‐way interactions between mitochondrial, nuclear haplotypes and dietary P:C ratios affected the strains' surface area of growth but not biomass. Intriguingly, strains did not increase their surface area and biomass when they had access to all diets on the buffet menu. Our findings have broad implications for our understanding of the effect of mitonuclear interactions on trait expression across diverse eukaryotic lineages.

## Graphical abstract

Three‐way interactions between mitochondrial, nuclear haplotypes and dietary P:C ratios affected the slime mould's surface area of growth but not biomass. Our findings have broad implications for our understanding of the effect of mitonuclear interactions on trait expression across diverse eukaryotic lineages.

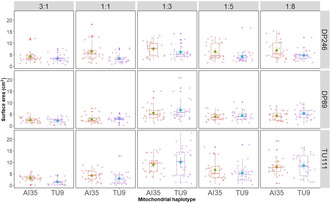

## 1 INTRODUCTION

More than two billion years ago, an endosymbiotic meeting between a prokaryotic cell and an archaeon led to the evolution of the eukaryotic cell (Gray et al., 1999; Sagan, 1967). Over evolutionary time, the prokaryote became the mitochondrion, the source of cellular energy in modern eukaryotes (Sagan, 1967). While the mitochondria had undergone significant changes from independent organisms to organelles, they retained their own genome. As a result, most modern‐day eukaryotic cells contain at least two independently evolving genomes: the nuclear genome and the mitochondrial genome (in the case of plants and algae, there is a third genome, that of the chloroplast; Bogorad, 1975). Together, nuclear and mitochondrial DNA are responsible for many aspects of eukaryotic cell function (Hill et al., 2019; Rand et al., 2004). In addition, epistatic interactions between the two genomes, referred to as mitochondrial–nuclear (hereafter, mitonuclear) interactions, are important for maintaining several essential biological processes (Cannino et al., 2007; Wolff et al., 2014). This includes the most important cellular biochemical process—oxidative phosphorylation (OXPHOS; Bar‐Yaacov et al., 2012; Friedman & Nunnari, 2014).

Until recently, it was assumed that the mitochondrial genome is strictly under strong purifying selection to ensure the integrity of the vital OXPHOS reaction (Avise, 1986; Rand, 2001). It was, therefore, generally presumed that mitonuclear interactions do not affect phenotype expression. However, this prevailing view has been challenged recently. The genetic variation in both mitochondrial and mitonuclear genomes significantly affects organism functioning across different levels—cell, tissue and whole organism (Hill et al., 2019; Shtolz & Mishmar, 2019; Wolff et al., 2014). Notable examples of mitonuclear genetic effect on phenotypic traits include longevity and reproductive outcomes in fruit flies (Patel et al., 2016; Zhu et al., 2014), metabolic rate and mating in seed beetles (Immonen et al., 2016), hearing impairment in mice (Johnson et al., 2001), mitochondrial physiology in nematodes (Chang et al., 2015) and differences in copy number of mitochondrial DNA in humans (Zaidi & Makova, 2019).

Mitochondria are central to metabolism in eukaryotes, converting oxidisable substrates in the diet into energy that drives cellular processes. Consequently, mitochondrial function is affected by the dietary environment (Simpson & Raubenheimer, 2009). Studies on fruit flies and mice show that the balance in dietary macronutrients, particularly proteins and carbohydrates, can affect the functioning of OXPHOS complexes and profoundly impact overall mitochondrial health (Pichaud et al., 2013; Solon‐Biet et al., 2014). In particular, protein concentration affects mitochondrial energy production and reactive oxygen species release (Ayala et al., 2007; Sanz et al., 2004; Solon‐Biet et al., 2014). Furthermore, a study on a large *Drosophila* mitonuclear panel found that three‐way G × G × E epistatic interactions between mitochondrial, nuclear DNA and dietary P:C ratio affect individual fitness (Mossman et al., 2016). Thus, mitonuclear interactions for phenotype expression in multi‐cellular eukaryotes are sensitive to the dietary environment.

Evidence for mitonuclear interactions with the environment affecting phenotype expression in single‐celled eukaryotes, such as the slime mould, are rare. In the last decade or so, the acellular slime mould *Physarum polycephalum*, a protist, has risen to prominence as a model organism to study decision‐making in the absence of a central nervous system (Beekman & Latty, 2015; Smith‐Ferguson & Beekman, 2020). *P. polycephalum* respond to environmental cues, sensing and moving towards food or away from toxic environments (Dussutour et al., 2010; Hatano, 1970; Kincaid & Mansour, 1978). Diet seems to affect mitochondrial biogenesis in slime moulds; for example, depriving *P. polycephalum* of glucose increases mitochondrial copy number (Oettmeier & Dobereiner, 2019). Furthermore, of particular relevance for the present study is the slime mould's ability to select the optimal protein‐to‐carbohydrate ratio when offered a range of diets (Dussutour et al., 2010). Dussutour et al. (2010) showed that a particular strain of *P. polycephalum* prefers a diet containing two‐part protein to one‐part carbohydrate (i.e. 2:1 P:C ratio). When given a choice of 11 different isocaloric diets that differed in P:C ratio (Dussutour et al., 2010), none of which contained the optimal P:C ratio, the organism was capable of distributing its biomass over different diets such that it obtained its preferred 2:1 P:C ratio (Dussutour et al., 2010). Whether the individual's genetic background mediates the diet effect on growth rate in slime mould remains unknown.

Given that diet affects the *P. polycephalum*'s growth rate, we used this organism to study the effects of mitonuclear interactions and diet on trait expression in single‐celled eukaryotes. Here, we utilised a mitonuclear panel consisting of six strains expressing two distinct mtDNA haplotypes (AI35 and TU9) and three nDNA haplotypes (DP246, DP89 and TU111). We used the hierarchy in the mating‐type locus (*matA*) of the haplotypes to induce uniparental mitochondrial inheritance in the crossing scheme (Meland et al., 1991; Moriyama & Kawano, 2003). The hierarchical order of *matA* allele of the haplotypes was: AI35 (matA2) > TU9 (matA11) > Tu111 (matA12) > DP89 (matA15) = DP246 (matA16). Thus, the haplotypes AI35 and TU9 served as the mitochondrial donors because they expressed dominant *matA* alleles than the nDNA haplotypes (Moriyama & Kawano, 2003). We further included the TU9 mitochondrial haplotype in our panel, which has a ~2 kb deletion mutation in the mtDNA that affects movement and growth in *P. polycephalum* (Moriyama & Kawano, 2003; Nagarajan‐Radha & Beekman, 2023). We also included the nDNA haplotype ‘TU111’, which harbours an ~18 kb extra‐chromosomal mitochondrial fusion‐promoting (mF) plasmid (Sakurai et al., 2004). Genetic crosses with the TU111 haplotype result in progenies that contain the mF plasmid along with the mtDNA from the respective mitochondrial donor (Kawano et al., 1991; Sakurai et al., 2004). For example, a cross between AI35 and TU111 yields progenies that express mtDNA from AI35 recombined with the mF plasmid from TU111. Essentially, the mF plasmid behaves as a ‘selfish’ element, ensuring its transmission into another cell lacking the plasmid. Yet, the plasmid has so far only been reported in laboratory strains, such as TU111, NG111, and JE8 (Kawano et al., 1991; Nomura et al., 2005; Sakurai et al., 2004), and is not known from any naturally occurring slime mould strains, suggesting that it might inflict a ‘cost’ onto its host. Thus, the mF plasmid‐carrying strains allowed us to test if the plasmid exerts any (negative) effects on the organism's growth rate.

We used biomass and surface area as proxies for growth because these are commonly used metrics of growth rate in *P. polycephalum* (Dussutour et al., 2010). We asked the following questions: (1) is the slime mould's growth rate affected by three‐way and two‐way interactions between mtDNA, nDNA haplotypes and diet; (2) do the slime mould strains exhibit differences in their ability to achieve higher growth rate when provided a choice of five experimental diets; and (3) does the mF plasmid negatively affect growth rate of the strains when given no choice in diet, and when given a choice. First, we studied growth rate differences across the strains when provided with each experimental diet in a *set* menu design. Then, we provided the strains with a *buffet* menu choice of all diets to measure their growth rate response. Given that mitonuclear interaction for phenotype expression is sensitive to the environment in *P. polycephalum*, we expected to see differences in growth rate across the strains in both set and buffet menu designs.

## 2 MATERIALS AND METHODS

### 2.1 Slime mould mitonuclear genetic panel

We used the mitonuclear genetic panel constructed in a previous study to measure mitonuclear interactions with diet for growth rate in the slime mould (Nagarajan‐Radha & Beekman, 2023). Briefly, the strains were created by crossing the haploid myxamoebae of the parental strains on a mating plate containing SM‐30 media, incubated at 24°C in the dark (Moriyama & Kawano, 2003). Diploid plasmodia emerging from the crosses were translocated to a fresh petri dish containing MEA growth media and incubated at 24°C in the dark (Moriyama & Kawano, 2003). Mature macroplasmodia of each strain were then grown in plastic containers (QUADRANT, 31 × 22 cm) filled with 2% w/v oatmeal agar media (made with 2% w/v powdered rolled oats and 1.5% w/v non‐nutrient agar), stored at 24°C with ~40% relative humidity in the dark (Nagarajan‐Radha & Beekman, 2023). We housed the strains on oatmeal agar media until the start of the experiment. We ensured the macroplasmodia had a constant supply of fresh oatmeal media by repeatedly transferring 5 × 3 cm macroplasmodia from old media onto new media every 48 h. We used three mitochondrial (cytochrome oxidase 1, cytochrome oxidase 3 and cytochrome b) and a nuclear (26s rRNA) gene sequences to deduce the mitonuclear haplotype of each strain. The sequences are available in NCBI's Genbank database with accession numbers—ON632012 to ON632035 (Nagarajan‐Radha & Beekman, 2023). We further confirmed the mF plasmid's presence in the strains expressing the TU111 nDNA haplotype by PCR amplifying DNA sequence located within the mF‐mtDNA recombined region (Nagarajan‐Radha & Beekman, 2023; Nomura et al., 2005).

### 2.2 Experimental diets varying in the protein‐to‐carbohydrate ratio

We designed five non‐isocaloric experimental diets that vary in protein: carbohydrate (P:C) ratios. The ratios were 3:1, 1:1, 1:3, 1:5 and 1:8, and the corresponding total calories in each diet were 194, 110, 65, 108 and 102 kcal respectively (Table S1). In these diets, we used mycological peptone (Oxoid Ltd.) as the protein source and Bacto malt extract (BD) as the carbohydrate source. Solid media were made with 1.5% w/v agar (GELITA Pty Ltd.). The precise amounts (g/L) of peptone, malt extract and agar were calculated based on each ingredient's percentage of protein and carbohydrate.

### 2.3 Effects of interactions between diet and mitonuclear genetic background on growth rate of slime mould—‘set menu’ design

In the first assay, we studied the growth rate of each of the six slime mould strains when grown on each of the five experimental diets—the *set* menu design. On each experimental day, an individual plasmodial fragment (2 × 2 cm) from the oatmeal agar media was transferred onto the centre of the petri dish (14.5 × 2 cm, Greiner Bio‐one) containing the particular experimental diet. We assayed up to four individual plasmodial fragments per strain per diet on each experimental day. The petri dishes were stored in a dark room maintained at 24°C and ~40% relative humidity to allow the growth of macroplasmodia for 48 h. We retrieved the Petri dishes from the darkroom at the end of the 48‐h growth period. Then, we photographed each petri dish (with a black background and standard light settings) using a digital camera (Canon corporation, EOS 1300D, EFS 18‐55 mm lens). A standard ruler (scale = 0.1 mm) was included in the background. Immediately after the photographs, we measured the wet biomass (to the nearest 0.001 g) of the macroplasmodia in each petri dish (Mettler Toledo PL403). We assayed 30 plasmodial fragments per strain per diet combination across 10 experimental days.

### 2.4 Experiment 2—Assaying growth rate of the slime mould strains when given a choice of all experimental diets—‘buffet menu’ design

In the second assay, we provided all experimental diets in a petri dish as a *buffet* menu option for the slime mould strains to determine if the slime moulds could achieve a higher growth rate when choosing from all diets. Each petri dish (14.5 × 2 cm) contained equal‐sized wedges of the five experimental diets, with a 2 × 2 cm piece of macroplasmodium placed in the middle of the petri dish (Figure S1). The macroplasmodia's search front always faced a marked point on the petri dish. We randomised the order in which the five experimental diets were arranged in each petri dish (clockwise from the marked point on the petri dish) to ensure that the macroplasmodia's decision was not affected by the diet the organism faced at the start of the experiment. As in our set menu experiment, we placed the macroplasmodia in the dark at 24°C for 48 h. The wet biomass on each diet wedge was individually estimated using the weighing balance to the nearest 0.001 g. We assayed 30 plasmodial fragments per slime mould strain across five experimental days.

### 2.5 Surface area data extraction from experiments 1 and 2

For our second measure, growth rate, we extracted the surface area of individual macroplasmodia grown on the experimental diets in both menu options. We estimated surface area of the macroplasmodia separately from each diet wedge in the buffet menu design. We analysed all photos using an online photo editing software, Photopea (www.photopea.com), to calculate the surface area of the macroplasmodia present on the experimental diets. First, we estimated the total surface area (*a*) of each photo (in cm^2^) using the ruler as a reference. Second, we calculated the total number of pixels in the photo (*b*), which included the total surface area of the petri dish. Third, the macroplasmodia were distinguished from the diet background using the quick selection and magic wand tools in the Photopea software. We then estimated the number of pixels in the selected area that shows the portion of the photo that the macroplasmodium occupied (*c*). Lastly, we calculated the surface area (cm^2^) of the macroplasmodia using the equation described below.

$$\text{Surface Area}\;\left({\mathrm{cm}}^{2}\right)=\frac{\text{Number of pixels of macroplasmodia}\;\left(c\right)\;}{\text{Total number of pixels in the photo}\;\left(b\right)}\times \text{Total surface area of photo}\;\left(a\right)$$

### 2.6 Statistical analyses

All analyses were run in the R statistical environment (R Development Core Team, 2020) using codes specified in the R package's documentation.

First, we analysed our set menu data using linear mixed‐effect regression (lmer) models in the lme4 package (Bates et al., 2015). Before we built statistical models, we estimated the magnitude and direction of the correlation between biomass and surface area across all combinations of strains and diets to inspect whether the response variables needed to be analysed separately. The overall correlation between biomass and surface area was 0.71 across all combinations of strain and diet (Pearson's correlation test, 95% confidence intervals = 0.68, 0.74, *p* < .0001), indicating that the variables should be analysed separately. Moreover, the response variables did not follow a normal distribution and so were square‐root transformed before the analyses. We then built separate lmer models for each response variable that included mtDNA haplotypes (two levels), nDNA haplotypes (three levels), experimental diets (five levels) and the interaction between these factors as fixed effects. The full model also included factors explaining the hierarchical structuring of the data—plasmodial replicate (30 levels) denoted as ‘plasmodia’ in the model, experimental replicate (180 levels—plasmodial replicates nested within strains), and interactions between random and fixed effects as random intercepts. We derived a final model by sequentially eliminating interaction terms (random and then fixed effects) that explained non‐significant (*p* > .05) variation in the response variables. We used a log‐likelihood ratio test to compare models. We selected the best model using the Akaike information criterion and *p*‐values from the model comparison test (Table S2).

The parameter estimates for fixed and random effects in the final models were calculated using the restricted maximum likelihood method. The numerator and denominator degrees of freedom and *p*‐value significance of each fixed effect were calculated using Type‐III F‐test with Kenward–Roger's approximation method. The standard deviation in the response variable attributable to each random effect was calculated from *summary* of the final model. In addition, the *p*‐value significance of each random effect was calculated using the *ranova* function in lmerTest package (Kuznetsova et al., 2017). Furthermore, we estimated the effect size (partial Cohen's *f* and its 95% confidence intervals) attributed to each fixed effect term using the *F‐to‐f* function in *effectsize* R package. The *F‐to‐f* function uses the *F*‐value, numerator and denominator degrees of freedom from the Type‐III *F*‐test results of the final models to calculate the Cohen's *f* effect size (Ben‐Shachar et al., 2020). And lastly, using the *emmeans* package, we tested for pairwise contrasts between all combinations of diets and strains to determine the negative effect of mF plasmid on the growth rate of slime mould.

Second, we tested the effect of calories on slime mould growth rate using a similar lmer approach as explained above. Here, the models included all fixed and random effect terms described above, with the exception of the categorical variable ‘diet’, which was replaced with the fixed covariate—‘calories’. The parameter estimates for fixed and random effects were estimated using the Type‐III *F*‐test with Kenward–Roger's method of approximation and restricted maximum likelihood method respectively. Lastly, we used the *effects* and *sjplot* R packages to extract the relationship between total calories and growth rate from the lmer models. We estimated the regression between total calories and growth rate parameters and the estimated marginal means (emmeans) of growth rate parameters adjusted for differences in total calories across all combinations of nDNA and mtDNA haplotypes. We further tested for significant pairwise contrasts across nDNA and mtDNA haplotypes using the *emmeans* package.

Lastly, to determine whether strains increase their biomass and surface area more or less on the buffet menu than the set menu, we first calculated the total biomass and surface area across all diets for each strain in the two menu designs. This resulted in 30 data points for each strain in each menu design. Given that the data points were non‐linear, we determined the relationship between the growth rate parameters using a polynomial regression analysis using *ggplot2* and *ggpmisc* packages in R. We generated a third‐order polynomial equation (with the associated coefficient of determination (*R* ^2^) and *p*‐value significance) for each pairwise comparison using *stat_poly_line* and *stat_poly_eq* functions in *ggpmisc* package (Wickham, 2016).

## 3 RESULTS

### 3.1 Slime mould's growth rate is affected by mitonuclear interactions and diet

**FIGURE 1:**
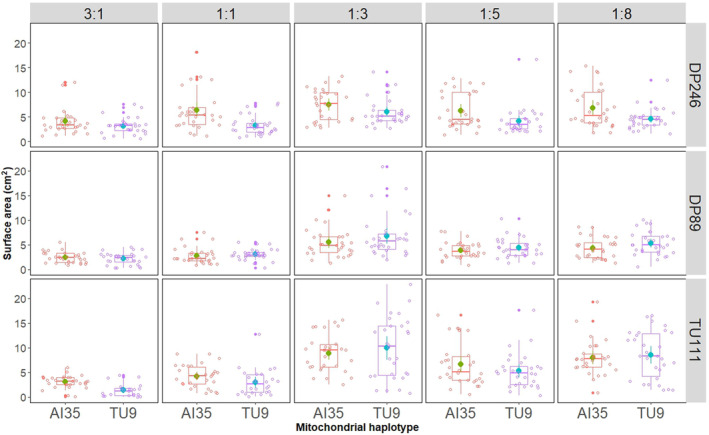
Three‐way interactions between mtDNA, nDNA haplotypes and dietary protein‐to‐carbohydrate (P:C) ratio affected the surface area of the slime mould strains. The strains expressed two distinct mtDNA haplotypes—AI35 and TU9 and three nDNA haplotypes—DP89, DP246, and TU111. Strains expressing the TU111 nuclear haplotype have a mitochondrial fusion (mF) plasmid integrated into its mtDNA. Vertical facets show P:C ratio of the experimental diets, and the horizontal facets show nDNA haplotypes. We estimated mean and standard error of surface area across 30 plasmodial replicates assayed for each mtDNA × nDNA × diet combination.

**FIGURE 2:**
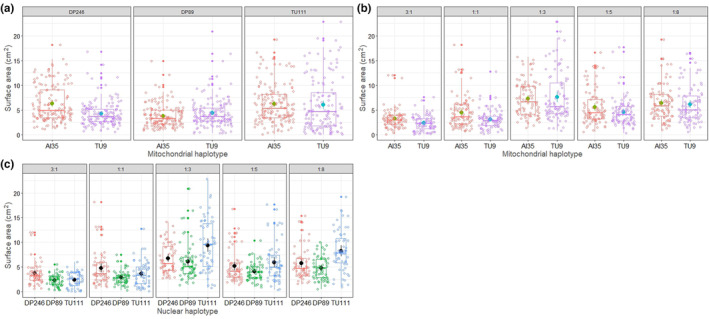
Effect of two‐way interactions between (a) mtDNA × nDNA haplotypes, (b) mtDNA × diet and (c) nDNA × diet on the surface area of growth across the slime mould strains. Data are pooled from 30 plasmodial replicates across combinations of mtDNA, nDNA haplotypes, and diet. There are 150 data points for each combination of mtDNA × nDNA in panel (a), 90 data points for each combination of mtDNA × diet in panel (b), and 60 data points for each nDNA × diet combination in panel (c).

**FIGURE 3:**
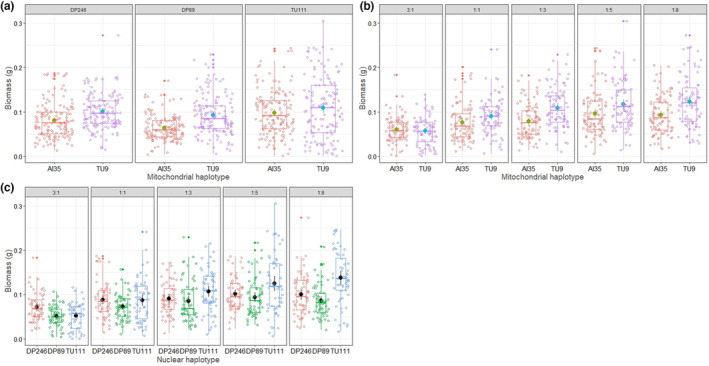
Effect of two‐way interactions between (a) mtDNA × nDNA haplotypes, (b) mtDNA × diet and (c) nDNA × diet on biomass of slime mould strains. Data are pooled from 30 plasmodial replicates across combinations of mtDNA, nDNA haplotypes and diet. There are 150 data points for each combination of mtDNA × nDNA in panel (a), 90 for each combination of mtDNA × diet in panel (b), and 60 for each nDNA × diet combination in panel (c).

The mitonuclear haplotype‐*by*‐diet three‐way interactions affected only the surface area (LMER analysis: mtDNA × nDNA × diet effect on surface area *F* = 2.1161, *p* = .033, Table 1a and Figure 1) but not strain biomass (LMER: *F* = 1.0275, *p* = .4138). We found significant mitonuclear interactions (mtDNA × nDNA) for both surface area (Table 1a and Figure 2a) and biomass (Table 1b and Figure 3a) of the strains. Two‐way interactions between genetic backgrounds and diet (i.e. mtDNA × diet and nDNA × diet) also affected the strains' surface area (Table 1a, Figure 2b,c) and biomass (Table 1b, Figure 3b,c). We found the effect size attributed to dietary P:C ratio to be the highest compared to genetic factors for surface area and biomass (Table 1a,b). Furthermore, we detected a significant effect of mF plasmid on surface area of the strains in diets—3:1, 1:1, 1:3, and 1:8 (Table 3a) and biomass in diets 3:1, 1:3, and 1:8 (Table 3b). The effect of mF plasmid on growth rate of the TU111 strains was not always negative. For example, the surface area and biomass of TU111 strains were comparatively lower on high‐protein diets but higher on low‐protein diets than other non‐mF plasmid strains (Table 3a,b).

**TABLE 1:** Results from linear mixed‐effect regression analysis of (a) surface area and (b) biomass estimates from the *set* menu design.

(a) Surface area model			
Fixed effects	*F*_*df*(num,den)_	*p*‐value	Cohen's *f* (95% CI)
mtDNA haplotype	13.469_1,29.11_	<.0005	0.68 (0.27, 1.08)
nDNA haplotype	22.5054_2,58.28_	<.0001	0.88 (0.56, 1.17)
Experimental diet	82.5944_4,116.34_	<.0001	1.69 (1.39, 1.96)
mtDNA × nDNA haplotype	14.769_2,58.28_	<.0001	0.71 (0.41, 0.99)
mtDNA × experimental diet	4.1496_4,570.44_	.003	0.17 (0.06, 0.24)
nDNA × experimental diet	10.323_8,570.9_	<.0001	0.38 (0.28, 0.45)
mtDNA × nDNA × diet	2.1161_8,571.19_	.033	0.17 (0, 0.22)
**Random effects**	**SD**	***p‐v***	
Plasmodial replicate	0.2684	.0001	
Experimental replicate	0.1319	.1405	
Plasmodial replicate × experimental diet	0.1433	.006	
Residual	0.5148		

(b) Biomass model			
Fixed effects	*F*_*df*(num,den)_	*p*	Cohen's *f* (95% CI)
mtDNA haplotype	22.5722_1,29.11_	<.0001	0.88 (0.45, 1.31)
nDNA haplotype	16.4867_2,58.27_	<.0001	0.75 (0.44, 1.03)
Experimental diet	66.1163_4,116.31_	<.0001	1.51 (1.23, 1.77)
mtDNA × nDNA haplotype	11.3469_2,58.27_	<.0001	0.63 (0.32, 0.89)
mtDNA × experimental diet	10.0752_4,571.03_	<.0001	0.27 (0.17, 0.34)
nDNA × experimental diet	8.3146_8,571.54_	<.0001	0.34 (0.23, 0.41)
**Random effects**	**SD**	***p***	
Plasmodial replicate	0.0344	<.0001	
Experimental replicate	0.0148	.1251	
Residual	0.0569		

*Note*: We built separate mixed‐effect models for each response variable in the lmer package. MtDNA haplotypes (two levels), nDNA haplotypes (three levels), experimental diets (five levels) and the interactions between these factors were included as fixed effects in the models. Furthermore, the plasmodial replicate (30 levels) and experimental replicate (180 levels—plasmodial replicate nested within strains), along with interactions between fixed and random effects, were modelled as random intercepts in the model. A final model was derived following the backward elimination of non‐significant fixed and random effect interaction terms. The parameter estimates for fixed effects in the final model were estimated using the restricted maximum likelihood estimation method and Type‐III *F*‐test with Kenward–Roger's approximation of degrees of freedom. In addition, we show the Cohen's *f* effect size and the 95% confidence intervals (CIs) estimated from the same final models. The *F*‐values, numerator and denominator degrees of freedom estimated from the Type‐III *F*‐test were used to calculate Cohen's *f* in *effectsize* package in R.

**FIGURE 4:**
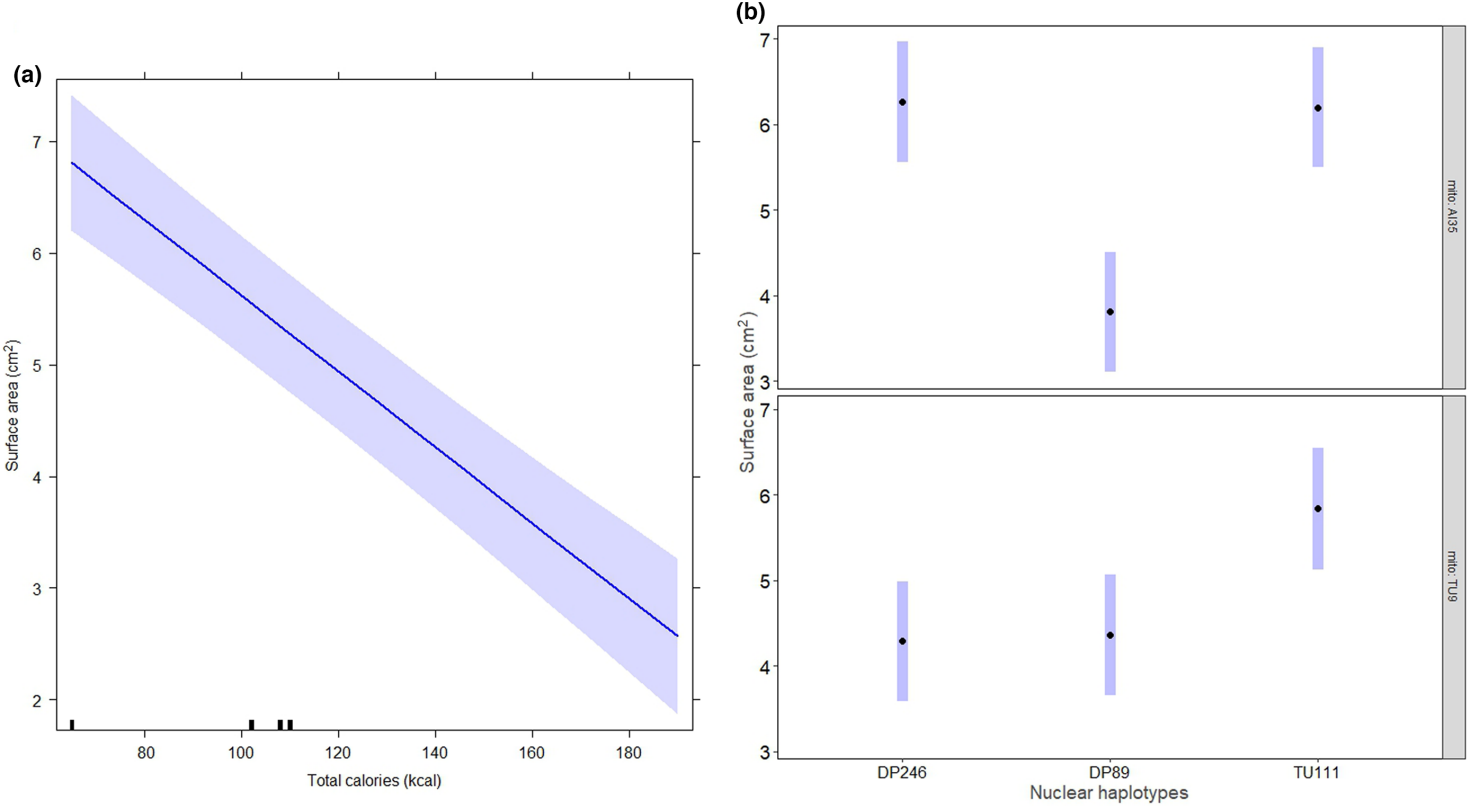
Effect of total calories on surface area of growth. (a) Effects plot from the mixed‐effect model showing linear regression line between surface area and total calories. And (b) pairwise differences in estimated marginal means and ±1 SE of surface area across mtDNA and nDNA haplotype combinations. The strain AI35.DP89 showed a significant difference in emmeans surface area with AI35.DP246 (difference = −2.45 ± SE = 0.33, *p* < .0001), and with AI35.TU111 (difference = −2.39 ± SE = 0.33, *p* < .0001) strains respectively.

**FIGURE 5:**
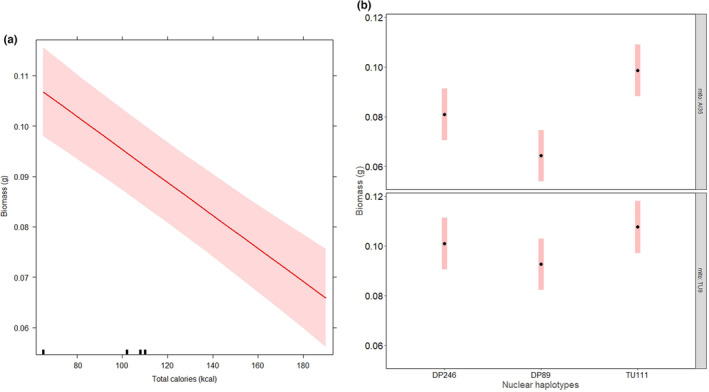
Effect of total calories on the biomass of strains. (a) Effects plot from the mixed‐effect model showing a negative relationship between biomass and total calories in the diet. And (b) pairwise differences in estimated marginal means and ±1 SE of biomass across the combinations of mtDNA and nDNA haplotypes. The strain AI35.DP89 showed a significant difference in emmeans biomass with AI35.DP246 (difference = −0.017 ± SE = 0.004, *p* = .0059), and with AI35.TU111 (difference = −0.034 ± SE = 0.004, *p* < .0001). In addition, we found that strain AI35.DP246 showed a significant difference in emmeans biomass with AI35.TU111 (difference = −0.018 ± SE = 0.004, *p* = .0027).

The total calories in the diet had a negative effect on surface area (Table 2a and Figure 4a) and biomass (Table 2b and Figure 5a) of slime mould strains. The strains achieved a high mean surface area and biomass on the 1:3 diet with the lowest calories (65 kcal), but their growth was low on the 3:1 diet with the highest calories (194 kcal). Thus, calorie restriction resulted in a higher growth rate across the strains. The effect size attributable to mitonuclear interactions was higher than calories in the diet for surface area and biomass (Table 2a,b). We further found the effect of calories on growth rate to be particularly significant in the strain AI35.DP89 compared to other strains (Figures 4b and 5b).

**TABLE 2:** Results from the mixed‐effect models testing the effect of calories and mitonuclear interactions on (a) surface area and (b) biomass of the strains.

(a) Surface area model			
Fixed effects	*F*_*df*(num,den)_	*p*	Cohen's *f* (95% CI)
mtDNA haplotype	11.11_1,29.026_	.002	0.62 (0.22, 1.01)
nDNA haplotype	26.433_2,58.003_	<.0001	0.95 (0.63, 1.25)
mtDNA × nDNA haplotype	14.714_2,57.996_	<.0001	1.11 (0.89, 1.34)
Total calories	152.825_1,122.639_	<.0001	0.71 (0.41, 0.99)
**Random effects**	**SD**	***p***	
Plasmodial replicate	0.2523	<.0005	
Plasmodial replicate × experimental diet	0.2294	<.0001	
Residual	0.5535		

(b) Biomass model			
Fixed effects	*F*_*df*(num,den)_	*p*	Cohen's *f* (95% CI)
mtDNA haplotype	25.2666_1,29.024_	<.0001	0.93 (0.49, 1.36)
nDNA haplotype	19.9143_2,58.002_	<.0001	0.83 (0.51, 1.12)
mtDNA × nDNA haplotype	8.3796_2,58.002_	<.0005	0.84 (0.64, 1.05)
Total calories	87.8198_1,123.205_	<.0001	0.54 (0.24, 0.8)
**Random effects**	**SD**	***p***	
Plasmodial replicate	0.0324	<.0001	
Plasmodial replicate × mtDNA haplotype	0.0149	.044	
Plasmodial replicate × experimental diet	0.021	<.0001	
Residual	0.061		

*Note*: The models included mtDNA haplotypes (two levels), nDNA haplotypes and their interactions as fixed effects in the models. We added total calories of each diet as a fixed covariate in the models. Furthermore, the models included plasmodial replicate (30 levels), experimental replicate (180 levels) and interactions between fixed and random effects as random intercepts in the model. We derived the parameter estimates for each fixed and random effect from the models using the restricted maximum likelihood estimation method and Type‐III *F*‐test with Kenward–Roger's approximation of degrees of freedom. Lastly, we estimated Cohen's *f* effect size (and 95% confidence intervals) of each fixed effect term using the *effectsize* package in R.

**TABLE 3:** The effect of mF plasmid on the (a) surface area and (b) biomass of slime mould strains across experimental diets.

(a) From lmer model analysing surface area estimate						
Contrasts	Diet	Estimate	Standard error	*Df*	*T* ratio	*p‐*value
AI35.DP246 – TU9.TU111	3:1	0.9731	0.157	637	6.208	<.0001
AI35.TU111 – TU9.TU111	3:1	0.7264	0.156	595	4.671	.0014
AI35.DP246 – TU9.TU111	1:1	0.8434	0.146	598	5.76	<.0001
TU9.DP246 – TU9.TU111	1:3	−0.5582	0.138	645	−4.06	.0176
AI35.DP89 – AI35.TU111	1:3	−0.6526	0.138	645	−4.746	.001
AI35.DP89 ‐ TU9.TU111	1:3	−0.6989	0.141	572	−4.96	.0004
AI35.DP89 – AI35.TU111	1:8	−0.7392	0.138	645	−5.376	<.0001
AI35.DP89 – TU9.TU111	1:8	−0.7682	0.141	572	−5.452	<.0001
TU9.DP89 – TU9.TU111	1:8	−0.5545	0.138	645	−4.032	.0195

(b) From lmer model analysing biomass estimate						
Contrasts	Diet	Estimate	Standard error	*Df*	*T* ratio	*p‐*value
AI35.DP246 – TU9.TU111	3:1	0.0902	0.0174	632	5.202	.0001
AI35.TU111 – TU9.TU111	3:1	0.0778	0.0172	590	4.524	.0027
TU9.DP246 – TU9.TU111	3:1	0.1001	0.0169	694	5.985	<.0001
AI35.DP89 – TU9.TU111	1:3	−0.0849	0.0157	563	−5.429	<.0001
AI35.DP246 – AI35.TU111	1:8	−0.0635	0.0152	644	−4.169	.0116
AI35.DP246 – TU9.TU111	1:8	−0.0926	0.0157	563	−5.917	<.0001
AI35.DP89 – AI35.TU111	1:8	−0.0869	0.0152	644	−5.708	<.0001
AI35.DP89 – TU9.TU111	1:8	−0.1161	0.0157	563	−7.415	<.0001
TU9.DP89 E – TU9.TU111 E	1:8	−0.0061	0.0152	644	−4.029	.0198

*Note*: Pairwise contrasts between strains and diet combinations were estimated from the final mixed‐effect models using *emmeans* package in R. Only the key contrasts with significant *p*‐values are listed in the tables.

### 3.2 Slime mould's growth rate did not improve when macroplasmodia were provided with a buffet of all experimental diets

**FIGURE 6:**
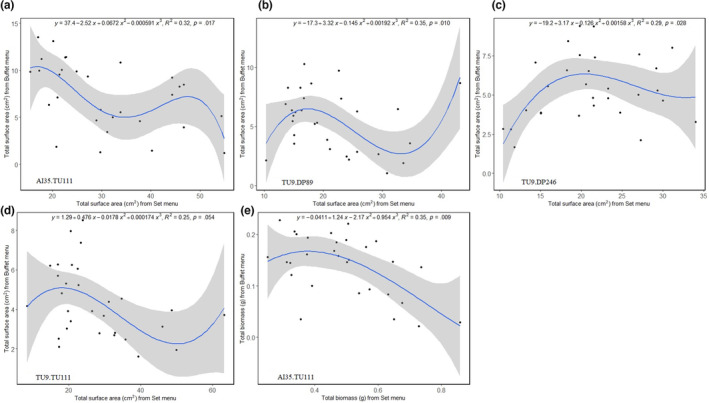
Polynomial relationship between the total surface area in (a) AI35.TU111, (b) TU9.DP89, (c) TU9.DP246 and (d) TU9.TU111 strains; and the total biomass in (e) AI35.TU111 strain, estimated from the set and buffet menu designs. The data were pooled across the diets, resulting in 30 data points for each strain in each menu design. The third‐order polynomial equation for each pairwise comparison was estimated using *stat_poly_line* and *stat_poly_eq* functions in *ggpmisc* package in R. Only pairwise comparisons with significant *p*‐value (<.05) for the coefficient of determination (*R* ^2^) are shown. Scale in the horizontal and vertical axes in each panel are adjusted to show the differences in magnitude and direction of relationship between the growth rate parameters in each slime mould strain.

Contrary to our prediction, we found the strains to grow slower on the buffet menu than on the set menu design (representative photos of slime growing on set and buffet menu designs are shown in Figure S2). The *R* ^2^ value for the polynomial relationship between the growth rate parameters estimated across the experimental designs was only significant in particular strains—AI35.TU111 (Figure 6a), TU9.DP89 (Figure 6b), TU9.DP246 (Figure 6c) and TU9.TU111 (Figure 6d) for surface area, and AI35.TU111 for biomass (Figure 6e). There was no clear negative effect of mF plasmid on the strains' growth in the buffet menu design (Figure 6a,d,e).

## 4 DISCUSSION

It has been challenging to convince evolutionary biologists that mitonuclear interactions are important in organismal biology because of the common assumption that the mitochondrial genome is under strong purifying selection (Rand, 2001). This traditional assumption needs to change because, increasingly, experimental evidence points to functional modifications encoded by mtDNA genetic variation. Moreover, coevolution between mitochondrial and nuclear protein‐coding genes, particularly those involved in oxidative phosphorylation, is required to support proper organismal functioning (further reviewed in Havird et al., 2019; Hill, 2019; Rand et al., 2004; Sloan et al., 2018). Another reason for the current sceptical take on mitonuclear ecology is that so far, evidence in support of the interaction between mitonuclear interaction and an organism's ecology is restricted to multi‐cellular eukaryotes, such as fruit flies (Hoekstra et al., 2013; Montooth et al., 2019; Mossman et al., 2016), seed beetles (Dordevic et al., 2017; Immonen et al., 2016), copepods (Barreto et al., 2018; Lima et al., 2019), birds (Morales et al., 2018; Trier et al., 2014), reptiles (Bar‐Yaacov et al., 2015), plants (Fujii et al., 2011) and humans (Zaidi & Makova, 2019).

Only a handful of studies have examined mitonuclear interactions in single‐celled eukaryotes, like yeast and fungi (Clergeot & Olson, 2021; Nguyen et al., 2023). Our findings that three‐ and two‐way interactions between the individual's mitonuclear genetic makeup and diet [and temperature (Nagarajan‐Radha & Beekman, 2023)] regulate growth in an acellular slime mould supports predictions of mitonuclear ecology theory from a new eukaryotic system and adds to the viewpoint that mitonuclear genetic effects on phenotypes are found across diverse eukaryotes. The phenotype output of each mtDNA haplotype was dependent on the nuclear genetic background and diet. The TU9 mtDNA haplotype harbouring a 2‐kb deletion mutation previously exhibited poor movement and growth across temperature treatments (Nagarajan‐Radha & Beekman, 2023). We did not find a negative effect of the TU9 mtDNA on growth rate across the diets suggesting that the haplotype might be sensitive only to temperature, but not the dietary environment. Furthermore, similar to findings from multi‐cellular systems, such as fruit flies, diet contributed a larger effect size on both growth parameters than the genetic factors (viz., mtDNA, nDNA and their interactions; Mossman et al., 2016; Nagarajan‐Radha et al., 2019). Thus, it is likely that diet affects mitonuclear interactions, and these G × G × E epistatic interactions, in turn, have an effect on phenotypes in diverse eukaryotes.

In contrast to our prediction, the strains could not optimise growth when offered all diets in the buffet menu compared to the set menu design. Previously, a study by Dussutour et al. (2010) showed that the HU554 × HU560 strain of *P. polycephalum* grows faster on a high protein, low carbohydrate diet (e.g. P:C = 2:1) and that the strain can distribute its biomass over different diets on offer to achieve optimal growth on their preferred P:C ratio (Dussutour et al., 2010). Most likely, the discrepancy between our study and that of Dussutour et al. can be explained by the difference in the way diet choice was set up. Dussutour's setup had 20 mm discs containing different P:C diets arranged on an agar media, whereas our setup had equal‐sized diet wedges placed on the petri dish. Furthermore, Dussutour's study used the HU strain (established at Hitotsubashi University, Japan and now popularly called the Southern Biological strain), which differs from the strains used in this study. We also think it is likely that slime mould strains react to different macronutrients in the diet. Dussutour et al. (2010) used glucose as the primary carbohydrate source and a mixture of calcium caseinate, whey protein and whole egg powder as protein sources, providing different amounts of calories than peptone and malt extract used in our diets. Lastly, we suspect the age of slime mould might affect diet choice. Contrary to the plasmodia used in our study, the macroplasmodia used in Dussutour's study were older. Older macroplasmodia might require high protein content for growth compared with young macroplasmodia. Age affects preferred P:C ratios in mice and humans (Senior et al., 2019, 2020).

Interestingly, we found no effect of the mF plasmid on slime mould's growth, regardless of the differences in macronutrient ratio and total calories in the diets. Mitochondrial plasmids exert strong negative effects on phenotypes in other organisms. For instance, mitochondrial plasmids are linked to cytoplasmic male sterility in plants (Chase & Pring, 1986; Erickson et al., 1986) and senescence in the fungus *Neurospora* (Bertrand et al., 1985). Interestingly, a plasmid‐like insertion in the mtDNA of *P. polycephalum* has previously resulted in immortality (Nakagawa et al., 1998). Despite the large size of the plasmid (~18 kb) found in the TU111 mtDNA and the additional cost required to replicate the extra amount of DNA for the mitochondrial DNA replication system, we found no apparent negative effect on the ‘strains’ growth rate. Interestingly, the plasmid‐carrying strain TU9.TU111 showed a higher growth rate on the 1:8 low‐protein diet than other strains. The lack of negative effect of carrying the mF plasmid begs the question of why the plasmid is not widespread. It could be that the mF plasmid is unlikely to be transmitted to spores during sexual reproduction, or it may not set a cost to the haploid plasmodial stage. These explanations remain speculative without further experimentation.

Despite our best efforts, our findings come with some caveats. First, since slime moulds, and more generally protists, have a higher mtDNA copy number and a higher frequency of inter‐mtDNA recombination events than animal cells (Gray et al., 1999, 2004), it was impossible to create isogenic strains. Yet, we found a strong G × G × E effect on growth rate, suggesting that the mitonuclear genetic effect on phenotypes is pervasive even in heterogenous genetic backgrounds and environments. While the mitochondrial and nuclear genomes of *P. polycephalum* have been sequenced (Glockner et al., 2008; Schaap et al., 2015; Takano et al., 2001), our previous sequencing effort yielded only ~7% of the total mitochondrial genome (4363 bp sequenced out of ~60,000 bp of *P. polycephalum* mtDNA; Nagarajan‐Radha & Beekman, 2023). Thus, the true genomic architecture of the strains in our panel remains unexplored. Furthermore, we could not extract genetic variance for growth rate of slime mould from our panel because it included only six strains that expressed mismatched mitonuclear genetic backgrounds. Studies that have reported genetic variance for a particular phenotypic trait in other model organisms have typically used larger genetic panels [e.g. 72 mitonuclear strains in a *Drosophila* study (Mossman et al., 2016)]. However, building such a large genetic panel of slime mould was impossible because of the limited number of strains available in the laboratory. Lastly, our diets varied in both P:C ratio and total calories, thus providing us with the means to statistically partition out the effect of each diet parameter on the growth rate of slime mould. While it is hard to quantify the relative contribution of calories and macronutrients for phenotype expression (Speakman et al., 2016), our findings, along with a study on mice, show that the P:C ratio affects organismal fitness more than calories in the diet (Solon‐Biet et al., 2014).

Regardless of the above caveats, our study provides a *proof‐of‐concept* for the predictions of mitonuclear ecology theory and further confirms that mitonuclear interactions with the dietary environment are essential for organismal functioning across diverse taxa. Studying the molecular mechanisms underlying growth rate response to G × G × E interactions, such as changes in gene expression and mitochondrial mRNA editing after exposure to different isocaloric diets in the slime mould, are interesting avenues for future studies to explore.

## AUTHOR CONTRIBUTIONS

**Venkatesh Nagarajan‐Radha:** Conceptualization (lead); data curation (equal); formal analysis (equal); project administration (lead); validation (lead); visualization (lead); writing – original draft (lead); writing – review and editing (lead). **Natalie Cordina:** Data curation (equal); investigation (equal); methodology (equal); software (lead); writing – review and editing (supporting). **Madeleine Beekman:** Conceptualization (supporting); formal analysis (supporting); funding acquisition (lead); methodology (supporting); project administration (supporting); resources (lead); writing – review and editing (equal).

## CONFLICT OF INTEREST STATEMENT

The authors declare that we have no known competing interests that could have appeared to influence the work reported in this paper.

### OPEN RESEARCH BADGES

This article has earned an Open Data badge for making publicly available the digitally‐shareable data necessary to reproduce the reported results. The data is available at https://doi.org/10.6084/m9.figshare.21286647.v2.

## Supporting information


Data S1:

## Data Availability

Raw data from the experiments are freely available in the Figshare repository under the DOI: https://doi.org/10.6084/m9.figshare.21286647.v2. Gene sequences are available in the NCBI Genbank server with the accession numbers: ON632012 to ON632035.
