# Supplementary material for: Diet and mitonuclear haplotype interactions affect growth rate in a slime mould
Source: Ecol Evol. 2023 Sep 4;13(9):e10508. doi: 10.1002/ece3.10508 (PMC10477482; doi:10.1002/ece3.10508)
Supplement: Supplementary file 1 — Data S1: [file ECE3-13-e10508-s001.docx]

***Diet and mitonuclear haplotype interactions affect growth rate in a slime mould***

*Venkatesh Nagarajan-Radha^1†^, Natalie Cordina^1^ and Madeleine Beekman^1^*

*^1^Behaviour, Ecology and Evolution Lab, School of Life and Environmental Sciences, The University of Sydney, Camperdown, NSW 2006, Australia.*

*^†^****Correspondence****: Dr Venkatesh Nagarajan-Radha -* [*venkatesh.nagarajan.radha@gmail.com*](mailto:venkatesh.nagarajan.radha@gmail.com)

***Electronic Supplementary Material***

***Supplementary Tables***

**Table S1**: The ingredients and respective quantities (g/L) used to prepare five experimental diets that varied in protein and carbohydrate ratio are listed. Mycological peptone served as the protein source, and Bacto malt extract was the carbohydrate source. Solid media was prepared with 1.5% w/v agar (81% carbohydrate and 6.2% protein). The proportion of protein and carbohydrate of each main ingredient was used to calculate the desired protein-to-carbohydrate ratios in the diet. Lastly, we used <https://www.omnicalculator.com/conversion/grams-to-calories> for converting grams of carbohydrates and protein into total calories.

| **3:1 diet** | Amount of ingredients in the diet (g/L) | Amount of protein in the diet (g/L) | Amount of carbohydrates in the diet (g/L) |
| --- | --- | --- | --- |
| Malt extract | 0 | 0 | 0 |
| Peptone | 35.55 | 35.55 | 0 |
| Agar | 15 | 0.9 | 12.15 |
| Total |  | 36.45 | 12.15 |
| Calories (kcal) | 194 | 145.8 | 48.6 |

| **1:1 diet** | Amount of ingredients in the diet (g/L) | Amount of protein in the diet (g/L) | Amount of carbohydrates in the diet (g/L) |
| --- | --- | --- | --- |
| Malt extract | 1.637 | 0 | 1.637 |
| Peptone | 12.887 | 12.887 | 0 |
| Agar | 15 | 0.9 | 12.15 |
| Total |  | 13.787 | 13.787 |
| Calories (kcal) | 110 | 55.15 | 55.15 |

| **1:3 diet** | Amount of ingredients in the diet (g/L) | Amount of protein in the diet (g/L) | Amount of carbohydrates in the diet (g/L) |
| --- | --- | --- | --- |
| Malt extract | 0.057 | 0 | 0.057 |
| Peptone | 3.169 | 3.169 | 0 |
| Agar | 15 | 0.9 | 12.15 |
| Total |  | 4.069 | 12.207 |
| Calories (kcal) | 65 | 16.276 | 48.83 |

| **1:5 diet** | Amount of ingredients in the diet (g/L) | Amount of protein in the diet (g/L) | Amount of carbohydrates in the diet (g/L) |
| --- | --- | --- | --- |
| Malt extract | 10.327 | 0 | 10.327 |
| Peptone | 3.595 | 3.595 | 0 |
| Agar | 15 | 0.9 | 12.15 |
| Total |  | 4.495 | 22.477 |
| Calories (kcal) | 108 | 17.98 | 89.9 |

| **1:8 diet** | Amount of ingredients in the diet (g/L) | Amount of protein in the diet (g/L) | Amount of carbohydrates in the diet (g/L) |
| --- | --- | --- | --- |
| Malt extract | 10.506 | 0 | 10.506 |
| Peptone | 1.932 | 1.932 | 0 |
| Agar | 15 | 0.9 | 12.15 |
| Total |  | 2.832 | 22.656 |
| Calories (kcal) | 102 | 11.328 | 90.62 |

**Table S2**: Results from mixed-effect model reduction analysis using Log-likelihood ratio test in R. The initial model included all fixed and random effects explained in the “statistical analysis” section of the main text. We employed a stepwise backward elimination approach to arrive at the final model. First, we tested if removing random effect interaction terms affected the model and then the interaction terms in fixed effects. We arrived at the final models for each response variable (surface area and biomass) separately. In the below table, the AIC value of the reduced model is provided within open brackets.

The initial full model for both variables:

*Full.model<-lmer (variable ~ mtDNA + nDNA + diet + mito:nuc + mito:diet + nuc:diet + mito:nuc:diet + (1|plasmo.replicate) + (1|plasmo.replicate:mito) + (1|plasmo.replicate:nuc) + (1|plasmo.replicate:diet) + (1|exp.replicate), REML=T, data=data)*

**Surface area model**

| **Model** | **AIC** | **P-value** |
| --- | --- | --- |
| Model1-(1\|plasmo.replicate:mito) | 1548.5 (1549.2) | 0.098 |
| Model1-(1\|plasmo.replicate:mito)- (1\|plasmo.replicate:nuc) | 1547.2 (1548.5) | 0.255 |
| Model1-(1\|plasmo.replicate:mito)- (1\|plasmo.replicate:nuc)-(1\|plasmo.replicate:diet) | 1552.9 (1548.5) | 0.016 |
| Model1-mito:nuc:diet-(1\|plasmo.replicate:mito)- (1\|plasmo.replicate:nuc) | 1709.9 (1672.9) | 0.027 |

**Final model for surface area**: *final.model<-lmer (sqrt(surface.area) ~ mtDNA + nDNA + diet + mito:nuc + mito:diet + nuc:diet + mito:nuc:diet + (1|plasmo.replicate) + (1|plasmo.replicate:diet) + (1|exp.replicate), REML=T, data=data)*

**Biomass model**

| **Model** | **AICc** | **P-value** |
| --- | --- | --- |
| Model1-(1\|plasmo.replicate:mito) | 2362.5 (2363.7) | 0.07 |
| Model1-(1\|plasmo.replicate:mito)- (1\|plasmo.replicate:nuc) | 2362.5 (2364.5) | 1 |
| Model1-(1\|plasmo.replicate:mito)- (1\|plasmo.replicate:nuc)-(1\|plasmo.replicate:diet) | 2364.5 (2366.1) | 0.5 |
| Model1-mito:nuc:diet-(1\|plasmo.replicate:mito)- (1\|plasmo.replicate:nuc)-(1\|plasmo.replicate:diet) | 2373.4 (2366.1) | 0.37 |

**Final model for biomass**: *Model<-lmer (sqrt(biomass) ~ mtDNA + nDNA + diet + mito:nuc + mito:diet + nuc:diet + (1|plasmo.replicate) + (1|plasmo.replicate:mito) + (1|exp.replicate), REML=T, data=data)*

***Supplementary Figures***


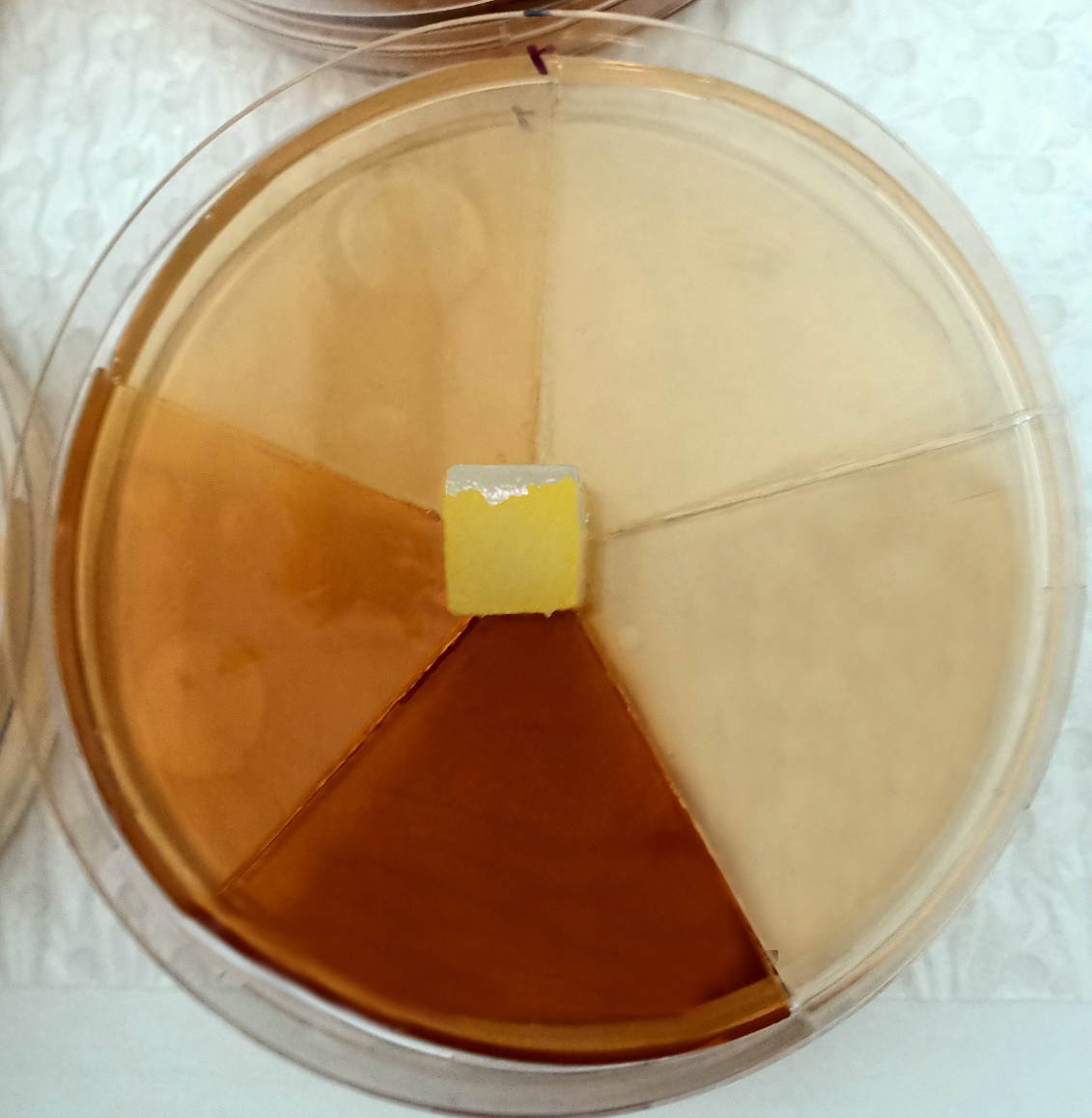


Marked Point on each petri dish

**Diet 4**

**Diet 5**

**Diet 1**

**Diet 2**

**Diet 3**

Slime mould search front always facing the marked point direction

**Figure S1**: Buffet menu design showing wedges from the five experimental diets arranged in a petri dish. A 2 cm × 2 cm slice of slime mould’s macroplasmodia was placed in the centre of the petri dish. The leading edge of macroplasmodia was placed towards a marked point on the petri dish. The position of diet wedges was randomised so that the slime mould faced different diets in each replicate.


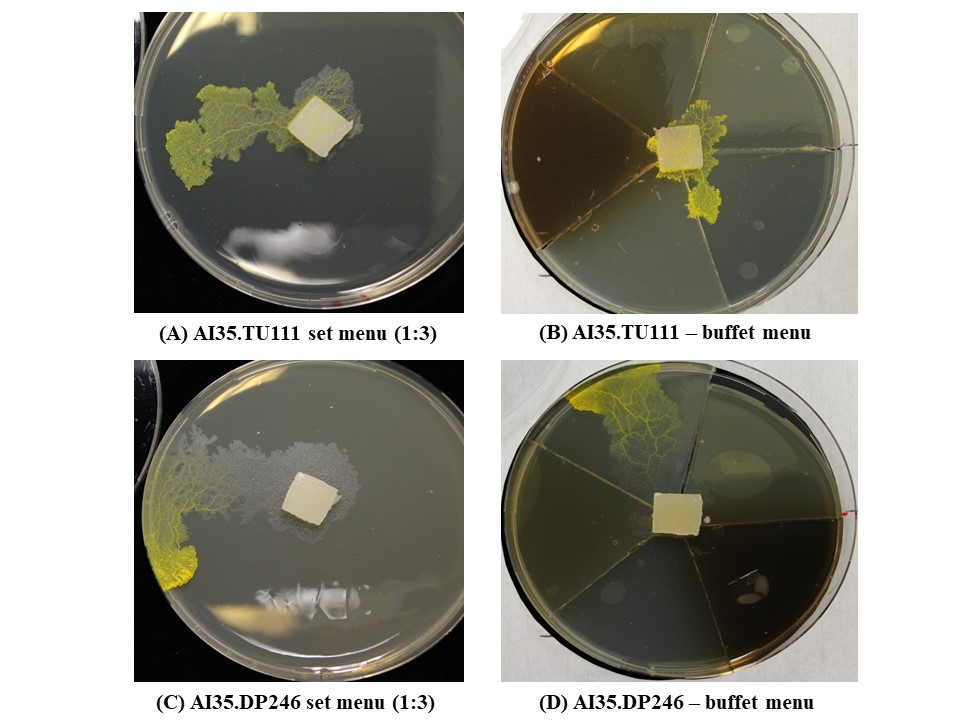


**Figure S2**: Representative photos of slime mould strain growing on the 1:3 set menu diet [(A) and (C)] and buffet menu designs [(B) and (D)] that had all diets. Strains generally grew faster on the set menu than on the buffet menu—the AI35.TU111 strain’s biomass and surface area from both designs were negatively correlated. On the other hand, the relationship between AI35.DP246 strain’s growth parameters were non-significant.
